# A panel of anti-influenza virus nucleoprotein antibodies selected from phage-displayed synthetic antibody libraries with rapid diagnostic capability to distinguish diverse influenza virus subtypes

**DOI:** 10.1038/s41598-020-70135-6

**Published:** 2020-08-07

**Authors:** Chung-Ming Yu, Ing-Chien Chen, Chao-Ping Tung, Hung-Pin Peng, Jhih-Wei Jian, Yi-Kai Chiu, Yueh-Liang Tsou, Hong-Sen Chen, Yi-Jen Huang, Wesley Wei-Wen Hsiao, Yong Alison Wang, An-Suei Yang

**Affiliations:** 1 Genomics Research Center, Academia Sinica, 128 Academia Rd., Sec.2, Nankang Dist., Taipei, 115 Taiwan; 2 Koo Foundation Sun Yat-Sen Cancer Center, Taipei, Taiwan

**Keywords:** Biological techniques, Biotechnology

## Abstract

Immunoassays based on sandwich immuno-complexes of capture and detection antibodies simultaneously binding to the target analytes have been powerful technologies in molecular analyses. Recent developments in single molecule detection technologies enable the detection limit of the sandwich immunoassays approaching femtomolar (10^–15^ M), driving the needs of developing sensitive and specific antibodies for ever-increasingly broad applications in detecting and quantifying biomarkers. The key components underlying the sandwich immunoassays are antibody-based affinity reagents, for which the conventional sources are mono- or poly-clonal antibodies from immunized animals. The downsides of the animal-based antibodies as affinity reagents arise from the requirement of months of development timespan and limited choices of antibody candidates due to immunodominance of humoral immune responses in animals. Hence, developing animal antibodies capable of distinguishing highly related antigens could be challenging. To overcome the limitation imposed by the animal immune systems, we developed an in vitro methodology based on phage-displayed synthetic antibody libraries for diverse antibodies as affinity reagents against closely related influenza virus nucleoprotein (NP) subtypes, aiming to differentiating avian influenza virus (H5N1) from seasonal influenza viruses (H1N1 and H3N2), for which the NPs are closely related by 90–94% in terms of pairwise amino acid sequence identity. We applied the methodology to attain, within four weeks, a panel of IgGs with distinguishable specificities against a group of representative NPs with pairwise amino acid sequence identities up to more than 90%, and the antibodies derived from the antibody libraries without further affinity refinement had comparable affinity of mouse antibodies to the NPs with the detection limit less than 1 nM of viral NP from lysed virus with sandwich ELISA. The panel of IgGs were capable of rapidly distinguishing infections due to virulent avian influenza virus from infections of seasonal flu, in responding to a probable emergency scenario where avian influenza virus would be transmissible among humans overlapping with the seasonal influenza infections. The results indicate that the in vitro antibody development methodology enables developing diagnostic antibodies that would not otherwise be available from animal-based antibody technologies.

## Introduction

Immunoassays based on sandwich immuno-complexes of capture and detection antibodies simultaneously binding to the target protein analytes have been powerful technologies in protein analyses. Recent developments in single molecule detection technologies enable the detection limit of the sandwich immunoassays approaching femtomolar (10^–15^ M)^1,2^. Applications of these immunoassays bring about tools in disease preventions/treatments, food safety assurances, immunogen detections and environmental contamination controls^3^. Consequently, the ever-increasingly broad applications in detecting and quantifying protein biomarkers, as well as the advancements in sensor technologies, are driving the needs of developing sensitive and specific antibodies as affinity reagents for immunoassays based on sandwich immuno-complexes.

The key components underlying the sandwich immunoassays are antibody-based affinity reagents, most of which are mono- or poly-clonal antibodies from immunized animals^4^, but there are substantial challenges frequently encountered in developing optimal animal-based antibodies useful in the sandwich immunoassays. The downsides of animal-based antibodies as affinity reagents are threefold: Firstly, the discovery and development timespan for animal antibodies requires up to 16–24 months^5^, which is much longer than the period critical in mitigating public health catastrophes, such as pandemic infectious disease outbreaks in humans. Secondly, animal B cell responses to an antigen are frequently focused on only a few immunodominant B cell epitopes of the antigen^6^, leading to limited choices of the animal antibodies as affinity reagents. Thirdly, even when animal antibodies become available as affinity antigens, the capability of these antibodies in distinguishing highly similar antigens is not guaranteed, and frequently, the end products could have the difficulty to distinguish virulent pathogen strains from their related but non-virulent ones.

Antibody discovery from phage-displayed synthetic antibody libraries is not subject to the limitations of animal immune systems^7–9^, and hence the shortcomings of animal-based antibody discoveries could frequently be overcome with the in vitro antibody discovery technologies. To streamline the development of antibody-based affinity reagents, we have designed and constructed a bank of GH (Generic Human) synthetic antibody libraries^7^, each of which contains more than one billion scFv variants suitable for high throughput assays^7,9–11^. The antibody discoveries against protein antigens with the GH synthetic antibody libraries have been highly successful in terms of attaining large number of non-redundant and highly specific antibodies for each of the target antigens^8,9,11–13^ with development timespan within a few weeks once the target antigen became available^9^.

The GH antibody technological platform could contribute mitigating probable influenza pandemic catastrophe by providing diagnostic antibodies capable of distinguishing subtypes of influenza virus type A (IAV). Influenza viruses can cross species barriers to infect diverse hosts due to rapid mutation, genetic drift and genome reassortment^14,15^, resulting in the emergence of novel influenza strains, such as H5N1 (Hong Kong) in 1997, H7N9 (China), H10N8 (China) and H6N1 (Taiwan) in 2013 and H5N6 (Hong Kong) in 2014^15,16^. Among the recently emerged influenza viruses, the highly pathogenic avian influenza H5N1 (Hong Kong) and H7N9 (China) are of particular concern. More than 400 human H5N1 infections since 1997 have been confirmed worldwide, and the mortality rate was higher than 60% for those cases who were immunocompetent^17,18^. Also, as of the end of May 2013, 132 human cases of infection with avian A influenza virus of the H7N9 subtype have been reported in China. 39 deaths have been known to be resulted from the infections^19^. In the worst scenario where highly virulent H7N9 or H5N1 strains were to emerge and transmit among human populations with naïve immunity, pandemics could arise without effective vaccine or anti-viral countermeasures available to the vast human populations. If the avian influenza virus (H5N1 or H7N9) outbreaks overlap with annual seasonal influenza virus infections (H1N1 and H3N2), there would be immediate challenges to rapidly distinguish the subjects infected with the pandemic avian influenza virus from the subjects infected with seasonal influenza virus strains. One way to confront the challenges would be developing antibodies as affinity reagents capable of distinguishing the subtypes of influenza virus.

There are unmet technical needs in developing diagnostic antibodies aiming to mitigate potential threats of influenza pandemics. Although sandwich immunoassays for the nucleoproteins (NPs) of influenza virus type A (IAV) and B (IBV) have been widely available as rapid influenza diagnostic tests^20–22^, the sensitivity of these tests are in the range of 40% to 70%^23,24^, partly due to the difficulty to cover increasingly diverse influenza strains^25^. More important, antibodies capable of distinguishing NPs from IAV subtypes, such as H1N1, H3N2, H5N1 and H7N9, had not been available, mostly because of the high similarities of the NPs of these IAVs in terms of pairwise amino acid sequence identity (~ 90%). A methodology of rapid and robust antibody development capacity would be of great value in responding not only to the influenza outbreak but also in managing other infectious disease outbreaks in humans and animals.

In this work, we assessed the GH in vitro antibody discovery methodology in terms of its capability in generating antibodies capable of distinguishing closely related NPs with sandwich immunoassays. Using the GH phage-displayed synthetic antibody libraries and an antibody discovering procedure designed to achieve our goal, we attained a panel of antibodies capable of differentiating the cognate NPs with pairwise amino acid sequence identity up to more than 90% and detection limit less than 1 nM with sandwich ELISA. The establishment of a diagnostic panel of antibodies with specificities and affinities sensitive enough to distinguish NPs from closely related influenza virus subtypes support the general methodology in rapid developing diagnostic antibodies for sandwich immunoassays of closely related analytes.

## Results

### Representative influenza nucleoproteins (NPs) derived from phylogenetic analysis of NP sequences in database were used as target antigens for anti-NP antibody discoveries

**Figure 1:**
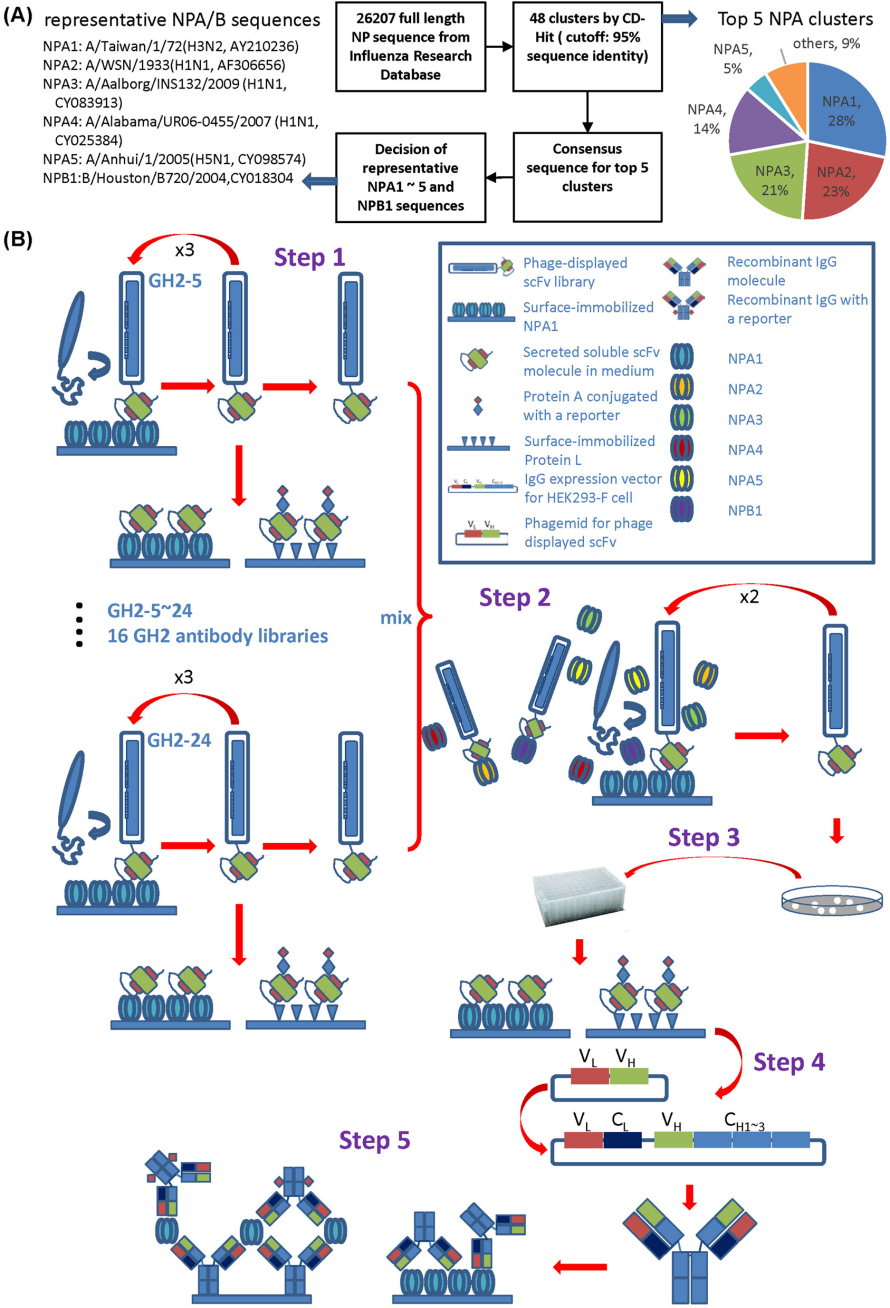
Representative NPs from the Influenza Research Data Base and the anti-NP antibody discovery procedure. (**A**) The flow chart shows the schematic procedure determining representative NPs. The details of the procedure are delineated in the main text and the phylogenetic tree of the NP sequences from Influenza Research Database is shown in Supplementary Figure S1. The sequences of the representative NPs are shown in Supplementary Figure S2. (**B**) The anti-NP antibody discovery procedure with phage-displayed synthetic antibody libraries is depicted schematically in 5 steps: Step 1 shows the phage display antibody selection procedure with multiple phage-displayed scFv libraries (results shown in Supplementary Figure S3) against the 6 representative NPs determined by the procedure shown in (**A**); Step 2 shows the additional two rounds of phage display selection to enrich the scFv libraries binding to the antigen immobilized on the solid surface by adding excessive amount of NPs other than the immobilized target NP; Step 3 shows the screening criteria for positive monoclonal scFvs (results shown in Fig. 2); Step 4 indicates the reformation and expression of the positive scFvs to monoclonal IgG1s; Step 5 depicts the binding immunoassay of the monoclonal IgG1 to the immobilized antigen on the right-hand side (results shown in Figs. 3, 4) and the sandwich immunoassay on the left-hand side (results shown in Fig. 5).

To assess the GH synthetic antibody library-based methodology in terms of its capability in generating antibodies capable of distinguishing NPs of influenza virus subtypes, we established a panel of NPs to represent, as broadly as possible, the NPs in nature as target analytes for the GH antibodies to differentiate. We clustered 26,207 influenza virus NP sequences from the Influenza Research Database^26^ with the software CD-HIT^27^ and the sequence identity threshold of 95% (Fig. 1A and Supplementary Figure S1). Out of the total of 48 clusters resulting from the clustering algorithm, the top 5 NPA (influenza A virus nucleoprotein) clusters encompassed 91% of the total NPA sequences and one NPB (influenza B virus nucleoprotein) cluster for all NPB sequences from the database (Fig. 1A and Supplementary Figure S1). This result is in agreement with the previously published phylogenetic analysis indicating that the NPA sequences can be phylogenetically grouped into only a few major clusters^28^. The consensus sequences of the top NPA and NPB clusters were used to search in the NCBI protein sequence database for representative NP sequences. The identities of the representative sequences (NPA1–NPA5 and NPB1) are shown in Fig. 1A and marked next to the phylogenetic tree in Supplementary Figure S1. The amino acid sequences and the pairwise sequence identities are delineated in Supplementary Figure S2A and S2B respectively. The phylogenetic tree of these representative NPs is shown in Supplementary Figure S2C. The results in Supplementary Figures S1 and S2 indicate that the NP of avian influenza virus H5N1 (NPA5) is similar to the NPs of seasonal influenza virus H1N1 (NPA2–4) and H3N2 (NPA1) by 90–94% pairwise amino acid sequence identities, posing the challenge upon the GH synthetic antibody library-based methodology to generate antibodies capable of distinguishing NPs of pairwise amino acid sequence identities above 90%.

We expressed NPA1–NPA5 and NPB1 as recombinant proteins respectively in *E. coli* harboring the chemically synthesized corresponding gene and purified the recombinant NPs to more than 95% purity for the following phage display antibody discovery procedure.

### An antibody discovery procedure was designed to develop a panel of anti-NP IgGs with diverse specificities to the representative NPs

To differentiate the influenza virus subtypes, we established a panel of antibodies with distinct binding patterns to the respective NP of the representative influenza viruses. A novel procedure schematically depicted in Fig. 1B was used for discovering antibodies for sandwich ELISA capable of detecting and distinguishing NPs from diverse influenza virus strains. Specifically, for each of the target NPs, the antibody discovery procedure in Fig. 1B started by 3 rounds of standard phage display selection^9^, using 16 GH synthetic antibody libraries^7^ respectively (Step 1 in Fig. 1B). The technical details of the construction of the general purpose GH phage-displayed synthetic antibody libraries and the standard procedure for phage-displayed antibody library selection and screening against the recombinant antigens have been documented in our previous publications^7–9^. The outcomes of the phage display selections are shown in Supplementary Figure S3. The selected phage-displayed libraries after 2 or 3 rounds of selection cycle with polyclonal scFv secretions in the culture media showing positive responses to the corresponding antigen with ELISA, as marked by the arrows in the panels of Supplementary Figure S3B, were expected to contain enriched candidate scFv populations binding to the corresponding antigen. These phage-displayed scFv libraries were mixed as input for another 2 rounds of phage display selection cycle, where the recombinant NPs other than the target NP immobilized on the solid surface were added in excess amount to the solution phase during the phage particle binding to the immobilized target NP (Step 2 in Fig. 1B). The purpose of these two additional selection rounds was to enrich the population of scFvs binding only to the target NP but not to the other NPs in the solution phase. Soluble monoclonal scFvs randomly selected from the output libraries of these two selection cycles were screened for binding to Protein A/L and to the respective NP with ELISA (Step 3 of Fig. 1B). The binding of a scFv to both Protein A and Protein L ensures that the scFv’s structure is stable and nativelike in solution^8,11^. The scFvs with positive binding signals to both Protein A/L and cognate NP were reformatted into IgGs with the human IgG1 framework. These IgG1s were expressed with mammalian expression system and purified with Protein A column (Step 4 of Fig. 1B), and then tested for antigen binding specificity and affinity with ELISA and sandwich ELISA (Step 5 of Fig. 1B).

### A panel of antibody-based affinity reagents with diverse specificities to the representative NPs were selected and screened from the phage-displayed synthetic antibody libraries

**Figure 2:**
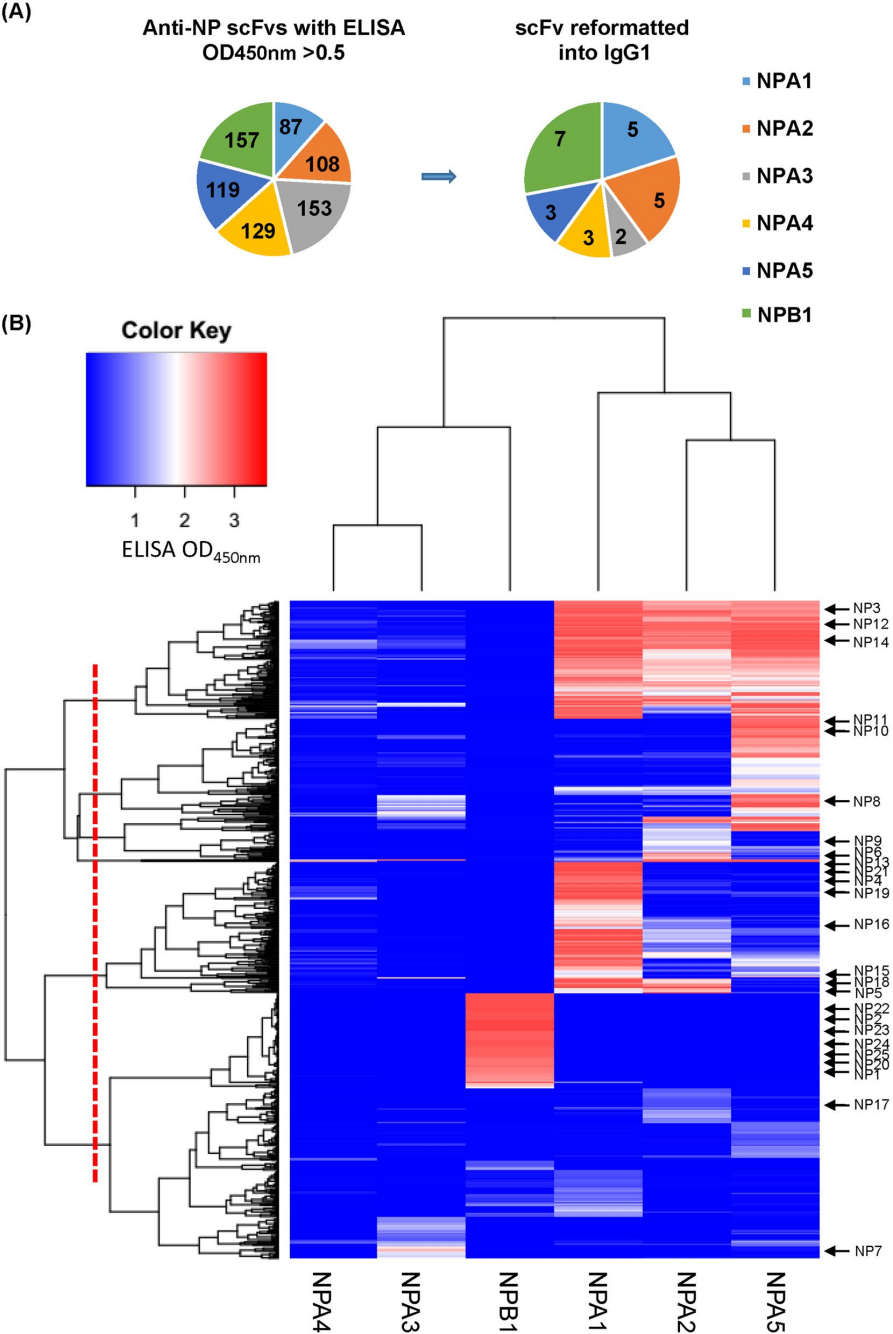
Summary of the anti-NP antibody discovery results. (**A**) The pie chart in the left-hand side shows the distribution of a total of 753 positive (ELISA OD_450nm_ > 0.5 binding to Protein A/L and to corresponding target NP) anti-NP monoclonal scFvs from the Step 3 of the antibody discovery procedure in Fig. 1B. The pie chart in the right-hand side shows the distribution of the 25 scFvs reformatted to IgG1 in the Step 4 of the antibody discovery procedure in Fig. 1B. These 25 IgGs were selected to form a panel of antibodies to represent the 753 scFvs according to the NP binding specificity groups as shown in (**B**) of this figure. The sequences of these 25 scFvs are listed in Supplementary Table S1. (**B**) The heat map shows the ELISA OD (450 nm) of the 753 anti-NP scFvs (y-axis) from the phage display selection cycles plotted against the 6 representative NPs (x-axis). The representative set of 25 scFvs are marked next to the heat map. The heat map and the clustering of the scFvs as shown by the dendrograms in the x-axis and y-axis were generated with the gplots package of the R software^7,35^. The experimental procedure for the ELISA measurement of the scFv-NP interactions is described in Methods.

A total of 753 positive monoclonal anti-NP scFvs (ELISA OD_450nm_ > 0.5 binding to Protein A/L and to corresponding target NP) were attained from the Step 3 of the screening procedure in Fig. 1B. The distribution of the 753 monoclonal scFvs versus the corresponding target NP used in the phage display selection is shown in the left pie chart in Fig. 2A. Each of these 753 monoclonal scFvs was tested for cross-binding to all the 6 NPs; the heat map in Fig. 2B shows the ELISA OD_450nm_ results for each of the 753 scFvs binding to the 6 NPs. The heat map is organized according to the grouping of the cross-binding pattern of the scFvs (y-axis of the heat map) to the 6 NPs (x-axis of the heat map). Based on the grouping of the scFv-NP binding pattern in Fig. 2B, we selected 25 scFvs to represent the major groups of the scFvs. These 25 scFvs are marked with arrows next to the heat map in Fig. 2B. The distribution of the 25 scFvs according to the target NP is shown by the right pie chart in Fig. 2A. The sequences of these 25 scFvs are listed in Supplementary Table S1. We reformatted the 25 scFvs into human IgG1s, which were expressed with the 293-F expression system and purified with Protein A column. The PAGE analysis for the expressed IgG1s is shown in Supplementary Figure S4.

### The anti-NP IgG1s bound to the recombinant NPs with diverse specificity and high affinity

**Figure 3:**
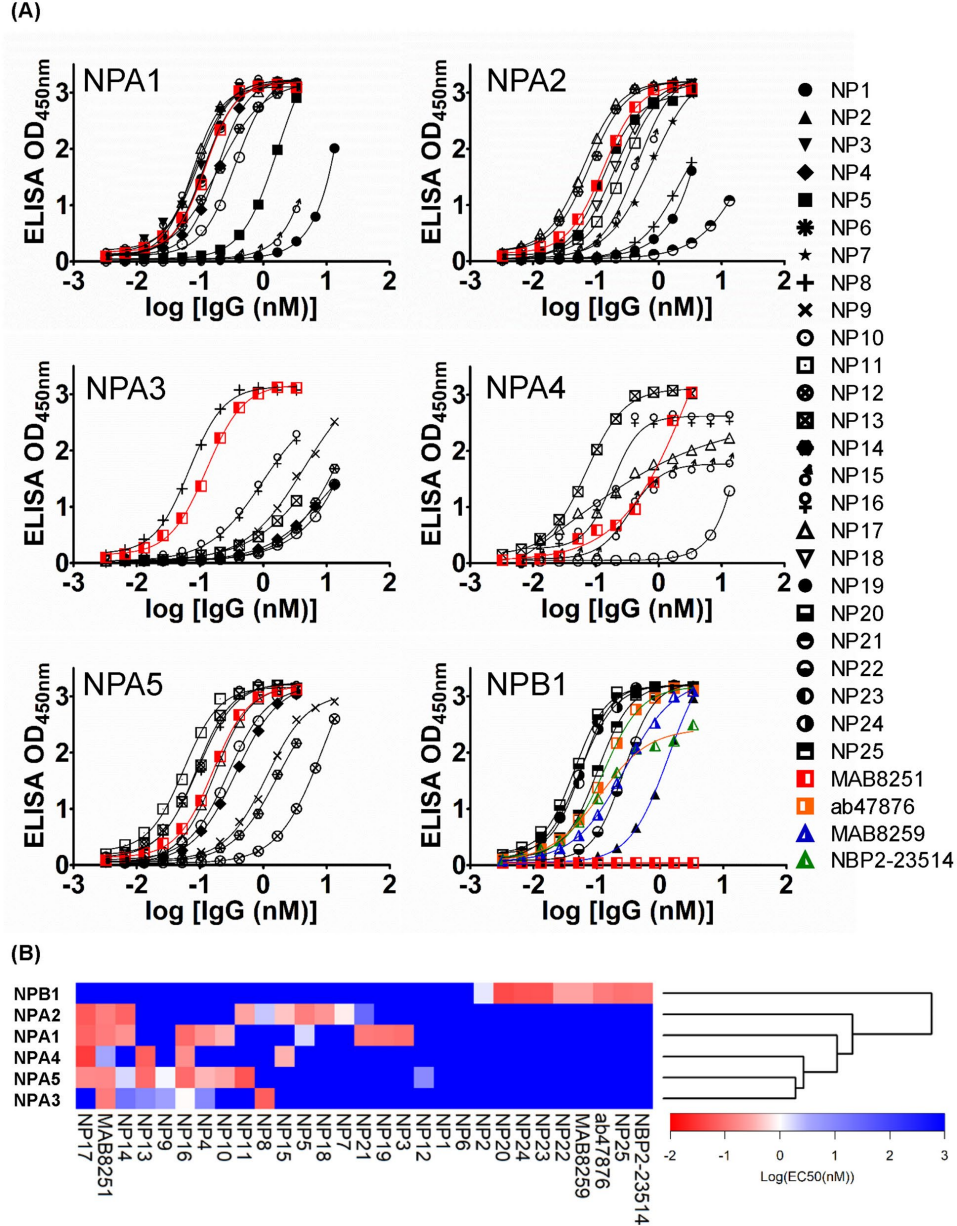
EC_50_’s of the anti-NP IgG1s reformatted from the 25 representative scFvs binding to the 6 representative recombinant NPs measured with ELISA. (**A**) The binding affinities as measured with ELISA (y-axis) for the anti-NP IgG1s binding to the NPs are plotted against the concentrations of the IgG1s (x-axis). The EC_50_’s of the binding curves were determined by fitting the binding curves with standard sigmoidal curve model with the software Prism, and are listed in Supplementary Table S2. (**B**) The heat map summarizes the specificities of the 25 anti-NP IgG1s (x-axis) in recognizing the 6 representative NPs (y-axis). The preparation of the IgG1s and the ELISA measurement of the IgG1-NP interactions are described in Methods. The heat map and the dendrogram were generated with the gplots package of the R software^35^.

The binding specificity and affinity of the 25 anti-NP IgG1s against the 6 NPs were measured quantitatively in terms of half maximal effective concentration EC_50_^7,9^, and were compared with those of the commercially available mouse monoclonal anti-NP antibodies as positive controls. As shown by the binding curves in Fig. 3A, for which the quantitative EC_50_’s are listed in Supplementary Table S2 and the binding specificities to the 6 NPs are summarized in Fig. 3B, the antibodies with the highest affinity binding to the corresponding recombinant NP with sub-nanomolar EC_50_’s were derived from the GH synthetic antibody libraries with the procedure shown in Fig. 1B without further affinity refinement (Fig. 3A,B; Supplementary Table S2), indicating that GH antibody platform could easily generate IgG1s with affinities and specificities superior to those of the control positive antibodies derived from animal immune systems against the target protein analytes. In comparison with the control positive antibody (MAB8251) with broad specificity to NPAs, NP16 and NP17 have broad specificity as the control positive antibody with comparable affinity (Fig. 3B). More importantly, the GH IgG1s with specific affinity to individual NPA1–NPA5 and NPB1, such as NP24-NPB1, NP3-NPA1, NP18-NPA2, NP15-NPA2/NPA4, NP8-NPA3/NPA2 and NP13-NPA4/NPA5 (Fig. 3B; Supplementary Table S2), enable affinity reagent-based profiling of NPs from unknown strains of IAV/IBV (see the two sections below).

### IAV subtype NPs in virus-infected MDCK cells were differentiated with the panel of anti-NP IgG1s

To test the capability of the panel of 25 anti-NP IgG1s in differentiating NPs from IAV and IBV, we carried out ELISA measurements to detect and differentiate the NPs expressed in MDCK cells infected by 5 vaccine strain IAVs and 1 vaccine strain IBV. Two groups of NPs were found in the 5 vaccine strain IAVs: the first group contains the NPs of A/Brisbane/59/2007(H1N1/H1B) and A/Brisbane/10/2007(H3N2/H3B) (background colored in orange in Supplementary Fig. 2B), which are identical in amino acid sequence and different from NPA4 by one residue (99.7% sequence identity; Supplementary Figure S2A, S2B (background colored in orange) and S2C); the second group contains the NPs of A/Wisconsin/67/2005(H3N2/H3W), A/California/07/2009(H1N1/H1S) and A/VietNam/1194/2004(H5N1/H5V) (background colored in yellow in Supplementary Fig. 2B), among which the pairwise amino acid sequence identities are different by at most 4 residues. The second group of NPs are similar to NPA2 with 96.9–97.1% sequence identity (Supplementary Figure S2A, S2B (background colored in yellow) and S2C). These two groups are similar to each other with 92.9– 93.3% pairwise sequence identity, as indicated in the part of Supplementary Figure S2B with background colored in blue. The NP of the vaccine strain IBV (B/Brisbane/60/2008(fluB)) is different from NPB1 by one amino acid residue (99.6% sequence identity; Supplementary Figure S2B (background colored in green)).

**Figure 4:**
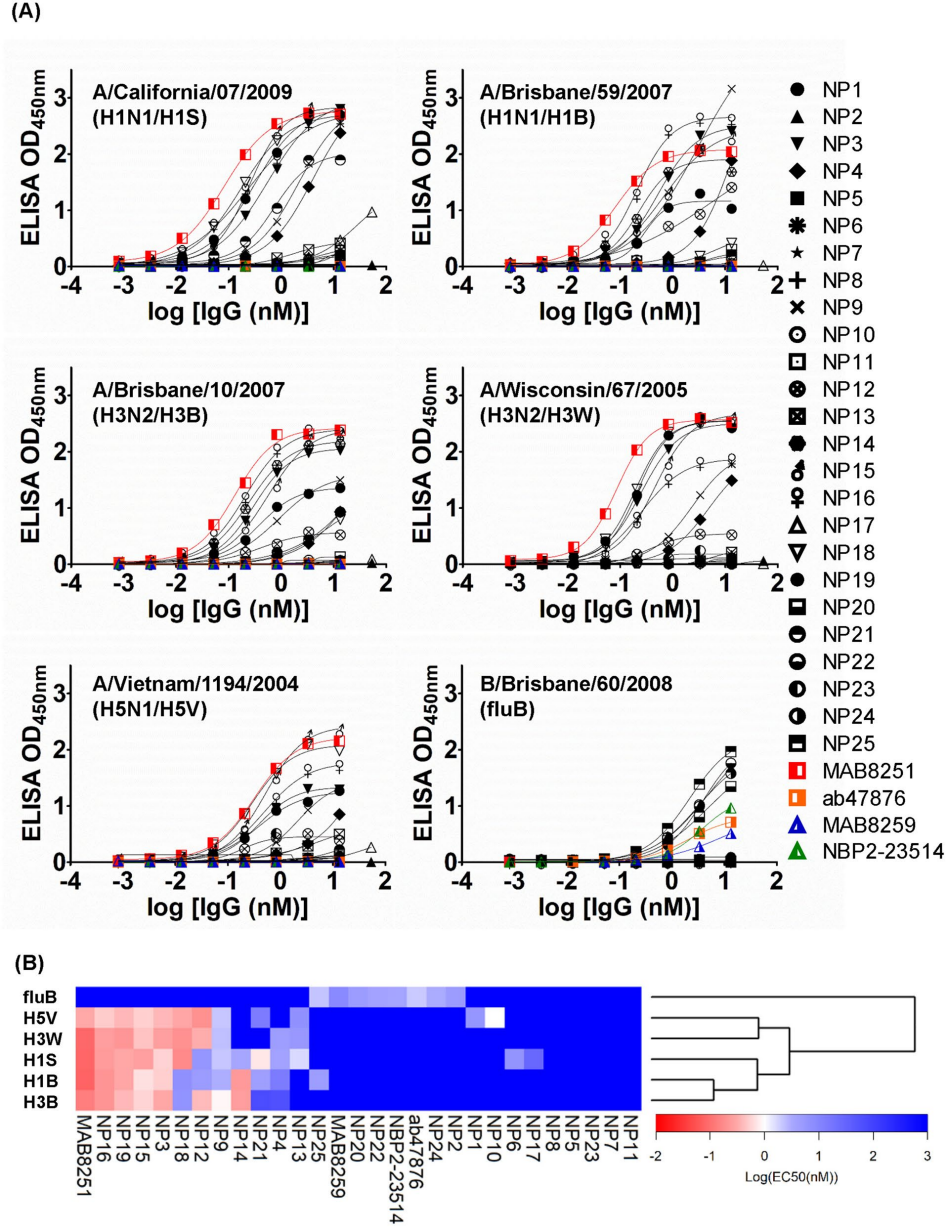
EC_50_’s of the anti-NP IgG1s reformatted from the 25 representative scFvs binding to the NPs in the virus-infected MDCK cells with ELISA. (**A**) The binding affinities as measured with ELISA (y-axis) for the anti-NP IgG1s binding to the NPs in the virus-infected MDCK cells are plotted against the concentrations of the IgG1s (x-axis). The EC_50_’s of the binding curves were determined by fitting the binding curves with standard sigmoidal curve model with the software Prism, and are listed in Supplementary Table S3. (**B**) The heat map summarizes the specificities of the 25 anti-NP IgG1s (x-axis) in recognizing the NPs from the virus-infected MDCK cells (y-axis). The preparation of the virus-infected MDCK cells and the ELISA measurements of the IgG1-NP interactions are described in Methods. The heat map and the dendrogram next to the heat map were generated with the gplots package of the R software^35^.

We tested the binding of each of the 25 anti-NP IgG1s to the NPs in immobilized MDCK cells pre-infected respectively with the 5 IAV and 1 IBV vaccine strains (Fig. 4A and Supplementary Table S3). Although the NPs expressed in virus-infected MDCK cells were not expected to completely resemble to the purified recombinant NPs in terms of NP-RNA complex and homo-polymer formation^29^, we nevertheless compared the results in Fig. 4 with those in Fig. 3. The EC_50_’s of the anti-NP IgG1s with the highest affinity binding to the corresponding NP in the virus-infected MDCK cells are comparable to those of the control positive antibodies (Fig. 4A), indicating that at least a subset of the 25 anti-NP IgG1s are able to bind to the NPs in the influenza virus-infected MDCK cells as effectively as the control positive antibodies (Fig. 4A and Supplementary Table S3). However, the specificities of the 25 anti-NP IgG1s against the NPs in MDCK cells infected by H1B and H3B are not exactly comparable with those against recombinant NPA4 (Fig. 3), the sequence of which is different from the NPs of H1B and H3B only by one amino acid residue (sequence identity 99.7%). Specifically, NP15 and NP16 consistently recognized recombinant NPA4 and the NPs in MDCK cell infected by H1B and H3B with high affinity, but similar consistency did not occur for NP13 and NP17, which recognized recombinant NPA4 with high affinity but failed to bind to the NPs in MDCK cell infected by H1B and H3B with observable affinity. In addition, NP3, NP9, NP12, NP14 and NP19, which did not have observable affinity to NPA4 (Fig. 3 and Supplementary Table S2), recognized the NPs in MDCK cell infected by H1B and H3B with observable affinity (Fig. 4 and Supplementary Table S3).

The anti-NP IgG1-NP binding patterns are able to differentiate closely related NPs expressed in MDCK cells (Fig. 4B). Not only the binding patterns of these anti-NP IgG1s to the NPs distinguish the NP of IBV from those of IAVs (Fig. 4B and Supplementary Table S3), the NPs from the subtypes of the IAVs are differentiable on the basis of the IgG1-NP binding patterns (Fig. 4B), which lead to correct grouping of the NPs of A/Brisbane/59/2007(H1N1/H1B) and A/Brisbane/10/2007(H3N2/H3B) with sequence identity of 100% and the NPs of A/Wisconsin/67/2005(H3N2/H3W) and A/Viet Nam/1194/2004(H5N1/H5V) with sequence identity of 99.3% (Supplementary Figure S2B). These two groups of NPs are different in sequence identity by 92.9–93.3%, which is correctly reflected in the grouping of the NPs based on the binding patterns of the anti-NP IgG1s to the NPs (the dendrogram of the y-axis in Fig. 4B). Still, the discrepancy between the grouping for A/California/07/2009(H1N1/H1S) based on the antibody binding patterns (the dendrogram of the y-axis in Fig. 4B) and the grouping based on the sequence identities (the phylogenetic tree in Supplementary Figure S2C) indicates the limitation in attempting to distinguish closely related NPs (~ 93% in sequence identity) with the antibody-NP binding profiles.

### Sandwich ELISA based on the panel of anti-NP IgG1s were capable of detecting and differentiating subtype NPs from lysed IAVs with detection limit of about 1 nM

The applicability of an antibody pair for sandwich immunoassays was determined with competition binding measurements, where one antibody was tested for binding to the immobilized analyte in the presence of the other antibody in the solution phase with excessive concentration to mask the epitope of the latter antibody on the analyte. If the binding of an antibody to the analyte is not affected by the presence of the competing antibody in excessively saturating quantity, the pair of antibodies is compatible as capture/detection antibody pairs in sandwich immunoassays. Supplementary Figure S5 shows the competition patterns of the panel of anti-NP antibodies binding to each of the 6 representative NPs. The competition measurements indicate that some pairs of the antibodies bound to non-overlapping epitopes in each of the 6 NPs, as indicated by the 0% competition antibody pairs (blue regions in the heat maps of the panels in Supplementary Figure S5) for each of the 6 NPs. The results suggested that the panel of the 25 anti-NP antibodies binding to the 6 representative NPs on at least 2 non-overlapping epitopes on each of the 6 representative NPs and that some pairs from the 25 anti-NP antibodies are suitable to differentiate the NPs with sandwich immunoassays.

**Figure 5:**
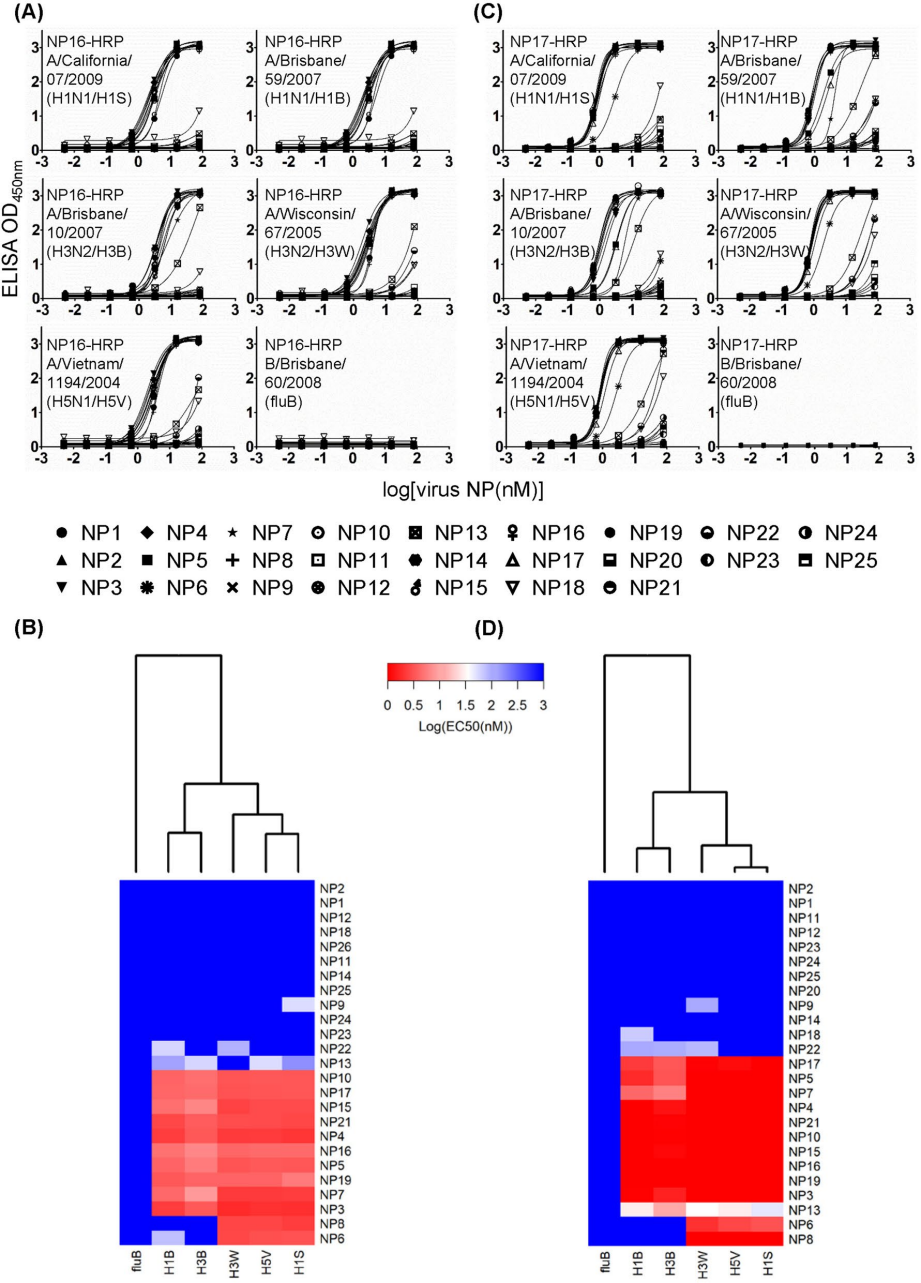
EC_50_’s of the NPs in lysed influenza virus measured by sandwich ELISA with the panel of 25 anti-NP IgG1s (NP1 ~ 25) as capture antibodies and the HRP-conjugated NP16 and NP17 as detection antibodies. (**A**) The 25 anti-NP IgG1s (NP1 ~ 25) were used as capture antibody respectively and the NP16 conjugated with HRP was used as detection antibody. The sandwich ELISA signals (y-axis) decrease with decreasing concentration of NP from lysed virus (x-axis). The EC_50_’s of the binding curves were determined by fitting the binding curves with standard sigmoidal curve model with the software Prism, and are listed in Supplementary Table S4A. (**B**) The heat map summarizes the specificities of the 25 anti-NP IgG1s (y-axis) in recognizing the NPs from the lysed influenza viruses (x-axis). The preparation of the HRP-conjugated IgG1s and the sandwich ELISA measurements of the IgG1-NP interactions are described in Methods. The heat map and the dendrogram were generated with the gplots package of the R software^35^. The descriptions of the (**C**) and (**D**) are the same as those of (**A**) and (**B**) respectively, except that the NP17 conjugated with HRP was used in place of NP16-HRP as detection antibody for the sandwich ELISA measurements. The EC_50_’s of the binding curves are listed in Supplementary Table S4B.

To test the profiling capabilities of the anti-NP antibodies in distinguishing closely related NPs from IAV subtypes with sandwich immunoassays, we measured the EC_50_’s of virus NPs from lysed IAVs using the sandwich ELISA with the capture and detection antibodies from the panel of the 25 anti-NP IgG1s. Again, the NPs from the lysed IAVs were not expected to completely resemble to the purified recombinant NPs and the NPs in the IAV-infected MSCD cells in terms of NP-RNA complex and homo-polymer formation^29^, hence the capture-detection pairs of antibodies used in the sandwich ELISA for quantitative detection of the NPs from the lysed IAVs had to be determined empirically. We used each of the 25 anti-NP IgG1s as capture antibodies and detected the NPs from the lysed IAVs using the HRP-conjugated NP16 and NP17 as detection antibody respectively (Fig. 5A,C respectively). NP17 and NP16 were selected because these two antibodies bind to NPs on non-overlapping epitopes (Supplementary Figure S5) with broad specificity against the representative NPs (Fig. 3). The results shown in Fig. 5A,C (Supplementary Table S4A and S4B) are highly similar, confirming that both NP16 and NP17 were competent as detection antibodies in recognizing the virus NPs from the lysed IAVs. NP17 and NP16 as both the capture and detection antibody can detect NPAs because of the formation of homo-polymer of the NPs^29^. The detection limit of the NPs from lysed influenza virus with the sandwich ELISA is on the order of 1 nM of virus NP. Moreover, the differentiation of the NPs from the subtypes of the IAVs based on the sandwich ELISA binding patterns (the dendrograms of the x-axis in Fig. 5B,D) is in close agreement with the phylogenetic analysis of these vaccine strain NPs (Supplementary Figure S2C). These results establish the usefulness of the sandwich ELISA with the panel of antibody-based affinity reagents as capture/detection antibodies in determining the quantity and subtype of NPs from lysed influenza viruses.

The NP from the lysed IAV resembled only to an extent to the corresponding NP expressed in virus-infected MDCK cells or *E. coli*. Comparing the results in Fig. 5 with those in Fig. 3, we found that NP13, NP15, NP16 and NP17 consistently recognized recombinant NPA4 and the virus NPs from H1B and H3B with high affinity. However, NP3, NP4, NP5, NP7, NP10, NP19 and NP21, which did not have observable affinity to NPA4 (Fig. 3 and Supplementary Table S2), recognized virus NPs from H1B and H3B with high affinity measured with the sandwich ELISA (Fig. 5 and Supplementary Table S4A and S4B). On the other hand, NP3, NP15, NP16 and NP19 recognized NPs from lysed virus and from virus-infected MDCK cells, but nevertheless, NP9, NP12 and NP14, which recognized NPs in H1B- and H3B-infected MDCK cells, did not have observable affinity to the corresponding NPs in the lysed IAVs. Although the NPs from the three preparations share common epitopes in the light that NP15 and NP16 recognized the corresponding NPs from the three different preparations, the recognition discrepancies described above also highlight the differences of the antigens due to the expression hosts.

## Discussion

The antibodies from the GH antibody discovery platform based on the phage-displayed synthetic antibody libraries in this work are able to distinguish highly related protein analytes through binding to the distinctive epitopes specific to each of the closely related protein analytes. As shown in Fig. 2, many scFv candidates had binding specificity only to recombinant NPA5 (NP of H5N1). Specifically, NP10 and NP11 in IgG1 form bound to NPA5 but also cross-reacted to NPA1 (NP of H3N1) and NPA2 (NP of H1N1) respectively (Fig. 3). Nevertheless, the combination of the binding specificities of NP10 and NP11 could distinguish NPA5 (NP10+ and NP11+), NPA1 (NP10+ and NP11−), and NPA2 (NP10− and NP11+) with the ELISA measurements in Fig. 3. The combination of these IgG1 specificities could distinguish the NP of avian influenza virus from those of seasonal influenza viruses with pairwise amino acid sequence identity up to 94.1%. Moreover, the sandwich ELISA measurements were more quantitatively reliable and most accurate in differentiating closely related NPs with pairwise sequence identities around 93% (Fig. 5). Sandwich immunoassays are anticipated to be more specific in terms of profiling closely related protein analytes in comparison with the binding immunoassays such as the experiments with the results shown in Figs. 3 and 4; the two immunoassay types are compared schematically in Step 5 of Fig. 1B. The reason for the superior performances of sandwich immunoassays is that, in sandwich immunoassays, the specificity of the protein analyte is characterized simultaneously with a pair of capture and detection antibodies binding to non-overlapping epitopes on the protein analyte, and as such the characterization of the analyte specificity in sandwich immunoassay is more stringent in comparison with the immunoassays that characterize the analyte specificity with only one antibody. The tradeoff of the stringency of analyte profiling with sandwich immunoassays is the prerequisite of a pair of antibodies binding to non-overlapping epitopes on the analyte with strong affinities. We have demonstrated in this work that the GH synthetic antibody libraries are particularly feasible in rapidly attaining suitable antibody pairs for sandwich immunoassays.

By contrast, animal-based antibody discovery technologies are limited by the animal immune systems. Animal antibodies providing the bulk of humoral immunity against antigens are generated from a population of antibody-secreting cells differentiated from the germinal center B cells selected by largely uncharacterized mechanisms for the immunodominant B cell epitopes of the antigens^6^. Unless we would have full insight into the function of the complete antibody repertories in animal immune systems and could artificially engineer and manipulate the antibody repertoires towards all available epitopes on the antigens, controlling the animal immune system in vivo to mount humoral immunity against all accessible epitopes, especially the less immunogenic ones, of the antigens is yet to be achievable. Alternatively, a panel of antibodies, such as the 25 anti-NP IgG1s in this work, with optimal affinities to diverse epitopes of the target antigens can be reliably derived from the synthetic antibody libraries designed and constructed to include antibody members with specificity and affinity for diverse antigens^7^. Antibody discoveries with the synthetic antibody libraries, as demonstrated in Fig. 2 of this work and other works^7–9^, are in vitro processes, where the distribution of the synthetic antibodies binding to diverse epitopes of the antigens is only governed by the design of the antibody libraries and the physical interactions of the antibodies and the antigens^7–9^. Consequently, as demonstrated in this work, these antibodies were able to recognize functional epitopes relevant to distinguishing closely related antigens with sequence similarity above 90%.

The timespan for the antibody discovery with the phage-displayed synthetic antibody libraries is much shorter than that with the monoclonal antibody technologies based on animal immune systems. For animal antibodies, several weeks or even months are necessary just to raise and immunize animals for required humoral immune responses to the antigens; hybridoma monoclonal antibody screening following sacrificing the immunized animals and harvesting the matured B cells could take another several weeks or months before the establishment of functional monoclonal antibodies against the antigens. In comparison, it took around 2 weeks after the availability of the antigens to complete the selection and screening of the scFvs from the phage-displayed synthetic antibody libraries; another 2 weeks were needed to express and purify the IgG1s reformatted from the selected scFvs in this work and in the work on anti-hemagglutinin antibody discoveries from the same set of phage-displayed antibody libraries^9^. Such in vitro antibody discovery procedures would be desirable when rapid development of antibody-based affinity reagents is crucial in controlling pandemic infectious disease outbreaks.

We have closely examined in this work the antibody-antigen specificities and affinities of a panel of anti-NP antibodies against NPs from three different sources. While the purified recombinant NPs used in antibody discoveries (Fig. 2) and EC_50_ measurements (Fig. 3) were free from RNA ligand to the NPs, the NPs in the IAV/IBV infected MDCK bound to indigenous RNA with unknown inhomogeneity in terms of RNA binding and homo-polymer formation^29^, and consequently, the accessible epitopes of the NPs in MDCK cells are expected to be different from those of the recombinant NPs harvested and purified from the *E. coli* expression system. Since the NP expression in the influenza virus-infected MDCK cells is virus strain-dependent and the expressed NP quantity cannot be quantitatively controlled, the EC_50_’s measured with the binding curves shown in Fig. 4 are understandably incomparable with those shown in Fig. 3. Moreover, although the NPs from the lysed influenza viruses are also known to be complexed with RNA in homo-polymer formation^29^, we found that the epitopes of the virus NPs resemble only to an extent to those of NPs in virus-infected MDCK cells based on the comparison of the ELISA profiling of the NPs in the virus-infected MDCK cells in Fig. 4 with the sandwich ELISA profiling of the NPs from the lysed influenza viruses in Fig. 5. Hence, it is critical to use the protein antigens that are most relevant to the practical applications for the antibody discovery process to ensure the applicability of the attained antibodies to recognize the protein analytes in practice.

In summary, for both in vitro and in vivo antibody discovery technologies alike, antibodies selected for the desired sandwich immunoassays are the most critical factor of attaining the goal of the application development. We demonstrated that a large number of antibodies selected from the GH synthetic antibody libraries bound to the 6 representative influenza NPs (5 from IAV strains and 1 from IBV strain) with corresponding affinities and specificities. Many of the optimal affinities of the selected antibodies for their corresponding NPs were below 1 nM in EC_50_ without the need for affinity maturation. The affinity level was comparable or superior to that of the positive control mouse antibody derived from murine immune system. The selected panel of antibodies together were diverse in specificities, capable of distinguishing NPs with pairwise amino acid sequence identities up to 93% with sandwich ELISA. The GH antibodies derived from the GH antibody libraries without further affinity maturation were used in sandwich ELISA to detect the corresponding NPs from lysed influenza viruses with detection limit of less than 1 nM of NP in specimen. This work demonstrates the feasibility of a general procedure in developing diagnostic antibodies that would be unavailable from animal-based antibody technologies.

## Methods

### Reagents

The mouse monoclonal anti-influenza nucleoprotein antibody MAB8251 (Millipore), MAB8259 (Millipore), ab47876 (Abcam), NPB2-23514 (1D8, Novus Biologicals), goat anti-mouse antibody-HRP (Cat. No. 12-349, Merck), and goat anti-human IgG Fc antibody (Cat. No. A80-304P, Bethyl Laboratories) were purchased. The 5 representative nucleoprotein (NP) of influenza A virus and 1 representative nucleoprotein (NP) of influenza B virus: NPA1 (AY210236), NPA2 (AF306656), NPA3 (CY083913), NPA4 (CY025384), NPA5 (CY098574), and NPB1 (CY018304) were prepared following the published procedure^30^. Specifically, the coding region of NP genes were optimized for *E. coli* expression and cloned into expression vector pET15b (NEB) linearized with NdeI and XhoI restriction enzymes; the recombinant NP protein contained a His_6_-tag and a thrombin cleavage sequence upstream to the NP sequence. These NP constructs were overexpressed in BL21 (DE3) cell with 0.5 mM IPTG induction at 16 °C. The purification of NP followed the previously reported procedures^31^. In brief, the NP recombinant protein expressed in *E. coli* was purified using Ni^2+^ charged Chelating Sepharose FF (for His_6_-tag binding), Hiprep heparin FF (for RNA-free NP binding), and Superose12 10/300GL columns (for size exclusive separation) with buffer contains 40 mM Tris, pH 7.5, 600 mM NaCl (GE Healthcare). To obtain the NP protein free of RNA, we applied RNaseA (20 μg/mL) to cell lysis of *E. coli,* followed by the purification procedures. Purified NP proteins were confirmed by SDS-PAGE.

### Cell lines

MDCK (Madin-Darby canine kidney, ATCC CCL-34) epithelial cells were cultured in MEM medium supplemented with NEAA (non-essential amino acids) (Cat. No. 10370021, Gibco), 2 mM l-glutamine, and 10% fetal bovine serum at 37 °C in a 5% CO_2_ humidified atmosphere incubator. 293-T cells (ATCC CRL-3216) were cultured in DMEM medium (11965092, Thermo Fisher Scientific Inc.) supplemented with 10% fetal bovine serum (10437028, Thermo Fisher Scientific Inc.), penicillin–streptomycin (100×, 15140122, Thermo Fisher Scientific Inc.). Suspension FreeStyle 293-F (293-F, R79007, Thermo Fisher Scientific Inc.) cells were cultured in serum free Freestyle 293 expression medium (12338001, Thermo Fisher Scientific Inc.) at 37 °C with shaking 110 rpm in 8% CO_2_ incubator (Thermo Fisher Scientific Inc.).

### Viruses

Influenza A viruses were attained from CDC in Taiwan: (1) H1N1 Brisbane (A/Brisbane/59/2007 (H1N1/H1B)); (2) H1N1 Swine (a recombinant virus NYMC X-181 derived from A/California/07/2009(H1N1/H1S)); (3) H3N2 Brisbane (A/Brisbane/10/2007(H3N2/H3B)); (4) H3N2 Wisconsin (A/Wisconsin/67/2005(H3N2/H3W)); (5) H5N1 Vietnam (a recombinant virus NIBRG-14 derived from A/VietNam/1194/2004(H5N1/H5V)); (6) Flu B (B/Brisbane/60/2008(fluB)). Viruses’ stocks were propagated in 10-day-old embryonic eggs’ allantoic cavities for 60 h and then harvested, concentrated by ultracentrifugation (25,000×*g* for 2 h) and resuspended in PBS. The virus titers and TCID50 (50% tissue culture infectious dose) were determined with cultured MDCK cells. In brief, the virus stocks were tenfold diluted by MEM-NEAA medium supplied with TPCK-treated trypsin (1 μg/mL) and 0.3% BSA (infection buffer). Diluted virus samples were incubated with PBS-washed MDCK cells (1 × 10^4^ cells per well in a 96-well plate) for 1 h. After absorption, the virus suspensions were removed and MDCK cells were washed by PBS twice. Infected MDCK cells were cultured in fresh infection buffer for either 3 days (H1N1 Swine, H3N2 Brisbane and H3N2 Wisconsin) or 5 days (H1N1 Brisbane, H5N1 Vietnam and Flu B). Survival MDCK cells were fixed with ice-cold methanol-acetone (1:1 (v/v)) and stained with 0.5% crystal violet and the TCID50 were calculated according to the Reed and Muench method.

### Characterization of the IgG1s derived from the selection and screening procedure with phage-displayed synthetic scFv libraries

The construction and characterization of the phage-displayed synthetic scFv libraries followed the same procedure, without modification, as described in the previous work^7,8^. The experimental procedures for panning the phage display libraries, selecting and screening of phage-displayed scFv binders, characterizing the scFvs binding to the cognate antigens and Protein A/L with ELISA, reformatting scFvs into IgG1s, expressing and purifying IgG1s, and determining EC_50_ for the antibody-antigen interaction with ELISA have been described in previous works^7,8,11,32–34^.

### IgG binding to NP from virus infected MDCK cells

MDCK cells (3 × 10^4^ cells/well) were seeded in 96-well plates for 16 h and washed twice with PBS prior to be infected by 100 × TCID50 viral solution. Infected MDCK cells were cultured for 24 h and then fixed with methanol-acetone (1:1 (v/v)). Staining procedure followed the previous publication^32^. In brief, a serial twofold diluted anti-influenza viral nucleoprotein IgG antibodies were used to detect viral nucleoprotein production with goat anti-human IgG-Fc antibody conjugated with HRP (1:5,000 dilution, A80-304P, Bethyl Laboratories) or goat anti-mouse antibody conjugated with HRP (1:1,000 dilution, 12-349, Millipore). Colorimetric measurements were carried out after the color development by adding TMB substrate (100 µL per well) to each well for 5 min before adding 1 N HCl (100 µL per well) to stop the chromogenic reaction. The absorbance at 450 nm was measured after Each concentration of diluted IgG was assayed with triplicate. EC_50_ was calculated as previously published^32^.

### Detection of NPs from lysed influenza virus with sandwich ELISA

Horseradish peroxidase (HRP) was conjugated to detection antibody with HRP Conjugation Kit (Abcam, ab102890). 200 μg of purified IgG was added to HRP mix with molar ratio IgG:HRP = 1:2 and the conjugation reaction was quenched according to manufacturer’s instruction. Sandwich ELISAs were carried out with 96-well Nunc plate, which was coated with purified capture IgG 1 μg per well at 4 °C overnight. NP from influenza virus was accessible in solution by lysing the virus with lysis buffer (PBS + 0.1% Tween-20 + 0.1% *N*-Lauroylsarcosine) for 1 h. The NPs from lysed viruses were quantified by running the query NPs through 12% NuPAGE Bis–Tris gels (Thermo Fisher Scientific Inc.) at 120 V for 3 h. The gels were stained with Coomassie brilliant blue, and the query NPs were quantified with Multi Gauge V3.0 software (Fujifilm) and the correlation of the Coomassie brilliant blue intensity versus the concentrations of the purified recombinant NPs. The quantified NPs from lysed influenza viruses were added to each well coated with capture antibody for one hour. After washing, 0.1 μg/mL HRP conjugated detection IgG (100 µL per well) was added to each well. The color was developed by adding TMB (ScyTek Laboratories, Inc.) (100 µL per well) to each well for 5 min before adding 1 N HCl (100 µL per well) to stop the chromogenic reaction before the absorbance at 450 nm was measured. EC_50_ was calculated as previously published^32^.

## Supplementary information

Supplementary Information 1.
