# Supplementary material for: A panel of anti-influenza virus nucleoprotein antibodies selected from phage-displayed synthetic antibody libraries with rapid diagnostic capability to distinguish diverse influenza virus subtypes
Source: Sci Rep. 2020 Aug 7;10:13318. doi: 10.1038/s41598-020-70135-6 (PMC7414213; doi:10.1038/s41598-020-70135-6)
Supplement: Supplementary file 1 — Supplementary Information 1. [file 41598_2020_70135_MOESM1_ESM.pdf]

Supplemental Information

A panel of anti-influenza virus nucleoprotein antibodies selected from phage-displayed synthetic antibody libraries with rapid diagnostic capability to distinguish diverse influenza virus subtypes

Chung-Ming Yu[1,2], Ing-Chien Chen[1,2], Chao-Ping Tung[1,2], Hung-Pin Peng[1], Jhih-Wei Jian[1], Yi-Kai Chiu[1], Yueh-Liang Tsou[1], Hong-Sen Chen[1], Yi-Jen Huang[1], Wesley Wei-Wen Hsiao[1], Yong Alison Wang[3], An-Suei Yang[1*]

[1] Genomics Research Center, Academia Sinica, Taipei, Taiwan.

[2] These authors contribute equally.

[3] Koo Foundation Sun Yat-Sen Cancer Center, Taipei, Taiwan.

[*] Correspondence should be addressed to: An-Suei Yang, Genomics Research Center, Academia Sinica, 128 Academia Rd., Sec.2, Nankang Dist., Taipei, Taiwan 115. Phone: +886-2-2787-1232 email: yangas@gate.sinica.edu.tw;

Supplementary Figures

Supplementary Figure S1. The phylogenetic tree of 26207 full length NP sequences from Influenza Research Database clustered with CD-HIT.

Supplementary Figure S2. Pairwise sequence identities, multiple sequence alignment and phylogenetic tree of the NPs in this work.

Supplementary Figure S3. The results of the antibody discovery procedure depicted in Figure 1B.

Supplementary Figure S4. SDS-PAGE analysis of the purified 25 anti-NP IgG1s.

Supplementary Figure S5. Competition of the anti-NP antibodies binding to the 6 representative NPs.

Supplementary Tables

Supplementary Table S1. The CDR sequences of the 25 representative scFvs indicated in Figure 2.

Supplementary Table S2. The EC_{50}'s (nM) derived from the sigmoidal binding curves of the 25 anti-NP IgG1s (first column from left) binding to the corresponding recombinant NP (first row from top) in Figure 3.

Supplementary Table S3. The EC_{50}'s (nM) derived from the sigmoidal binding curves of the 25 anti-NP IgG1s (first column from left) binding to the NPs in the influenza virus-infected MDCK cells (first row from top) in Figure 4.

Supplementary Table S4. The EC_{50}'s (nM) of the virus NPs (first row from top) derived from the sigmoidal binding curves in Figure 5 of the sandwich ELISAs with HRP-conjugated NP16 (Table S4A) and NP17 (Table S4B) as detection antibody and the 25 anti-NP IgG1s (first column from left) as capture antibodies.

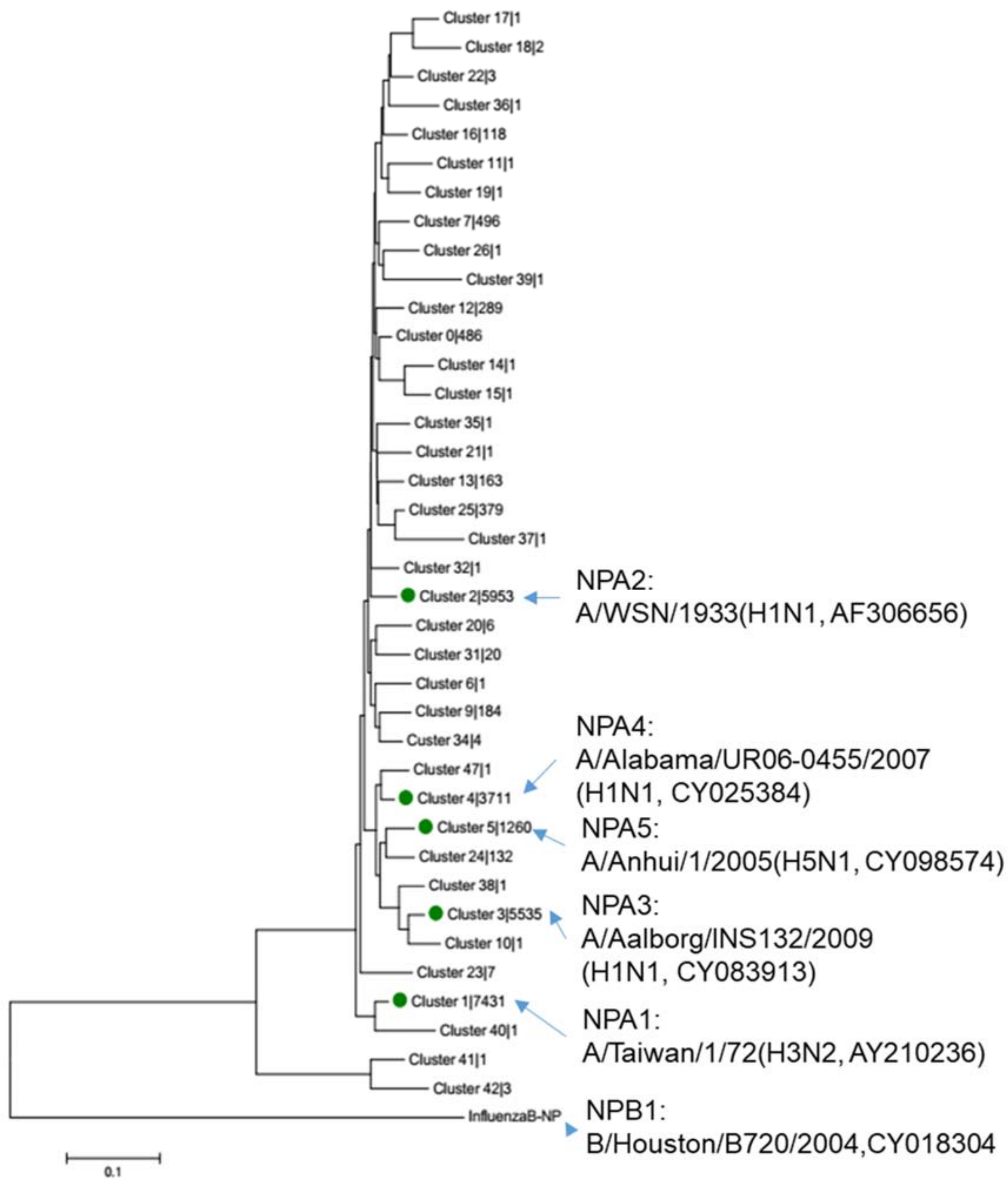

Supplementary Figure S1. The phylogenetic tree of 26207 full length NP sequences from Influenza Research Database clustered with CD-HIT. The 5 representative NPA clusters and the NPB cluster are marked next to the phylogenetic tree with the identities of NPA1~5 and NPB1.

A

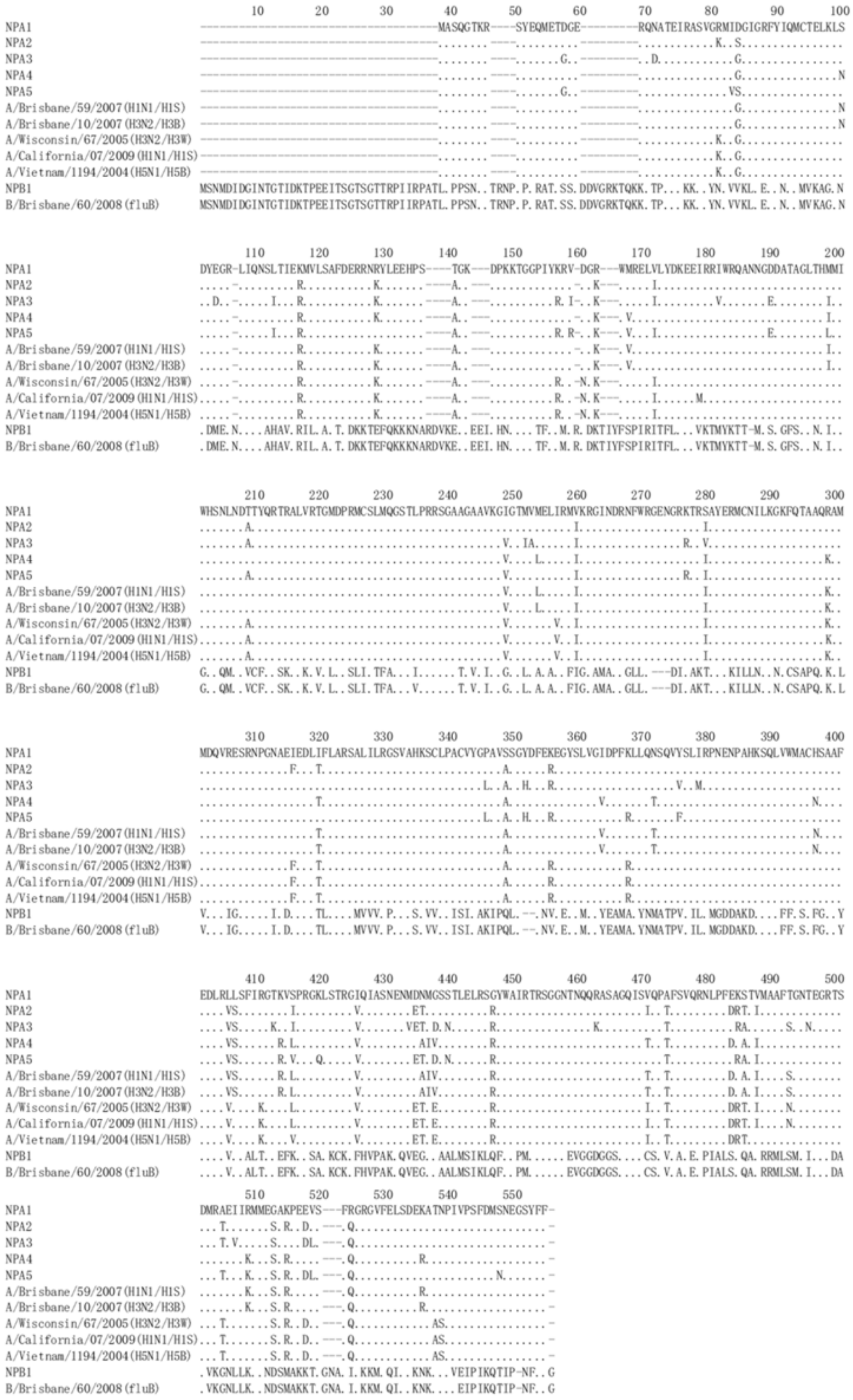

B

NP antigen	NPA1	NPA2	NPA3	NPA4	NPA5	A/Brisbane/59/2007(H1N1/H1B)	A/Brisbane/10/2007(H3N2/H3B)	A/Wisconsin/67/2005(H3N2/H3W)	A/California/07/2009(H1N1/H1S)	A/Vietnam/1194/2004(H5N1/H5V)	NPB1	B/Brisbane/60/2008(fluB)
NPA1		93.5%	89.7%	92.5%	90.5%	92.3%	92.3%	91.5%	91.3%	91.9%	34.2%	34.4%
NPA2	93.5%		92.7%	94.3%	94.1%	94.1%	94.1%	97.1%	96.9%	97.1%	34.8%	34.9%
NPA3	89.7%	92.7%		89.9%	94.3%	90.1%	90.1%	91.5%	91.3%	91.7%	34.6%	34.8%
NPA4	92.5%	94.3%	89.9%		92.1%	99.7%	99.7%	93.3%	93.1%	93.1%	35.1%	35.3%
NPA5	90.5%	94.1%	94.3%	92.1%		91.9%	91.9%	92.9%	92.7%	93.1%	34.4%	34.6%
A/Brisbane/59/2007 (H1N1/H1B)	92.3%	94.1%	90.1%	99.7%	91.9%		100.0%	93.3%	93.1%	92.9%	35.3%	35.5%
A/Brisbane/10/2007 (H3N2/H3B)	92.3%	94.1%	90.1%	99.7%	91.9%	100.0%		93.3%	93.1%	92.9%	35.3%	35.5%
A/Wisconsin/67/2005 (H3N2/H3W)	91.5%	97.1%	91.5%	93.3%	92.9%	93.3%	93.3%		99.7%	99.3%	34.0%	34.2%
A/California/07/2009\ (H1N1/H1S)	91.3%	96.9%	91.3%	93.1%	92.7%	93.1%	93.1%	99.7%		99.1%	34.0%	34.2%
A/Vietnam/1194/2004 (H5N1/H5V)	91.9%	97.1%	91.7%	93.1%	93.1%	92.9%	92.9%	99.3%	99.1%		34.2%	34.4%
NPB1	34.2%	34.8%	34.6%	35.1%	34.4%	35.3%	35.3%	34.0%	34.0%	34.2%		99.6%
B/Brisbane/60/2008 (fluB)	34.4%	34.9%	34.8%	35.3%	34.6%	35.5%	35.5%	34.2%	34.2%	34.4%	99.6%	

C

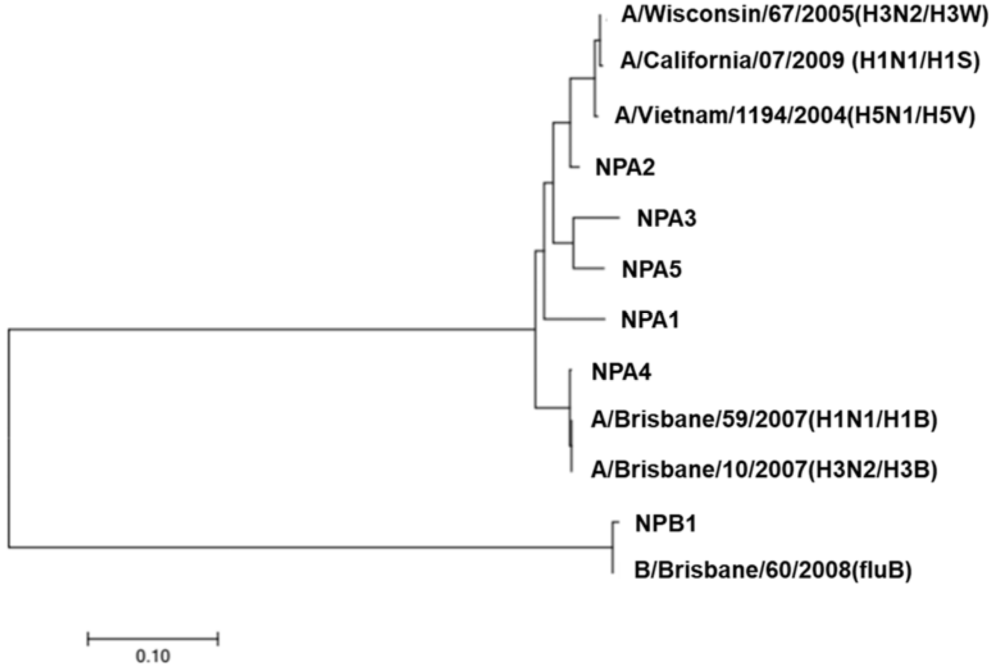

Supplementary Figure S2. Pairwise sequence identities, multiple sequence alignment and phylogenetic tree of the NPs in this work. (A) The multiple sequence alignment of the NP sequences of NPA1~5, NPB1 (see Figures 1~3 of the main text) and the NPs from the vaccine strains of IAV and IBV (see Figures 4~5 in the main text) is shown. (B) The pairwise sequence identities of the NP sequences in the panel (A) of this figure are shown in the matrix. (C) The phylogenetic tree of the NP sequences in the panel (A) of this figure is shown in this panel.

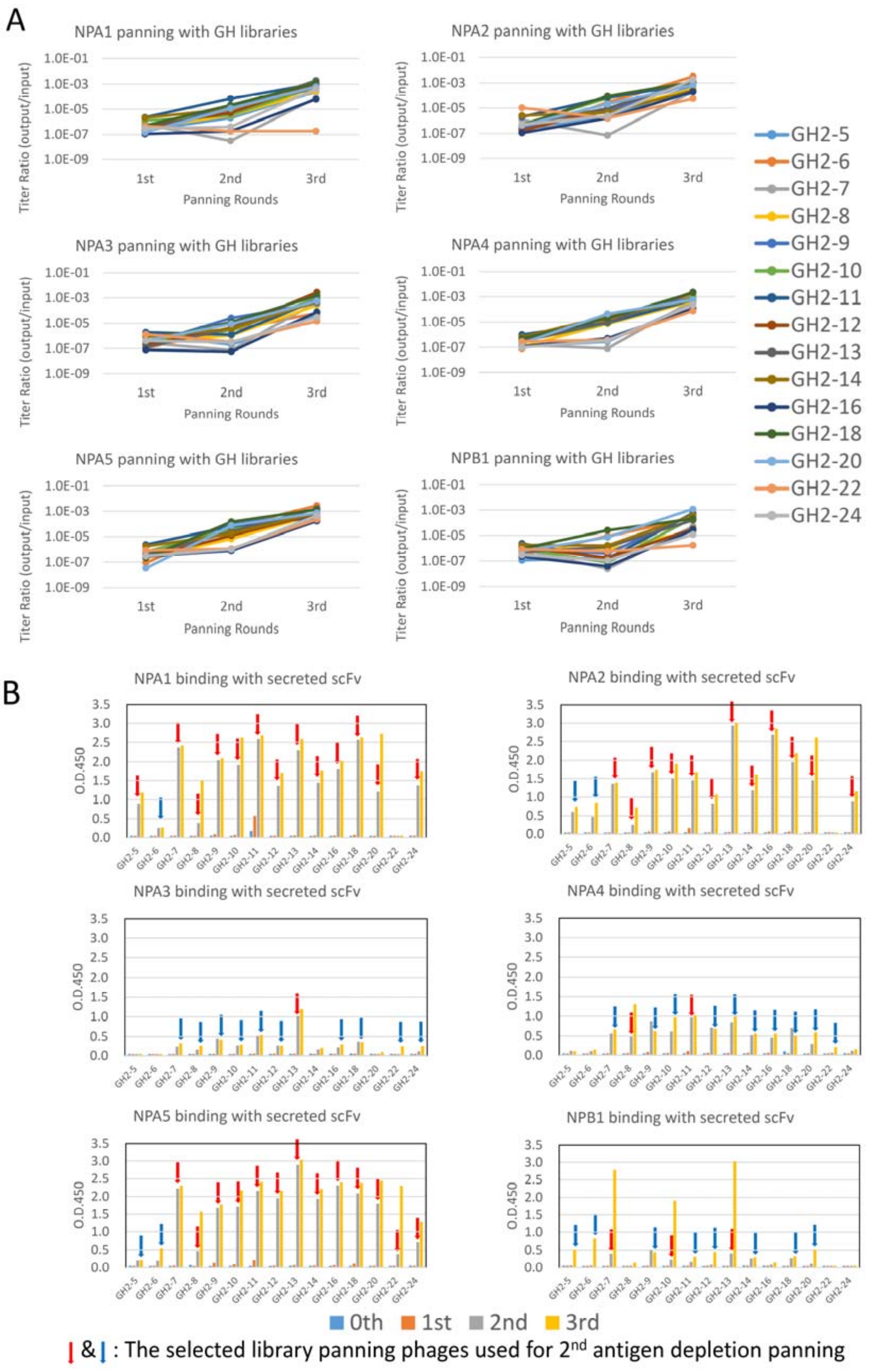
A
NPA1 panning with GH libraries
NPA2 panning with GH libraries
NPA3 panning with GH libraries
NPA4 panning with GH libraries
NPA5 panning with GH libraries
NPB1 panning with GH libraries
Titer Ratio (output/input)
1.0E-01
1.0E-03
1.0E-05
1.0E-07
1.0E-09
1st
2nd
3rd
Panning Rounds
GH2-5
GH2-6
GH2-7
GH2-8
GH2-9
GH2-10
GH2-11
GH2-12
GH2-13
GH2-14
GH2-16
GH2-18
GH2-20
GH2-22
GH2-24
B
NPA1 binding with secreted scFv
NPA2 binding with secreted scFv
NPA3 binding with secreted scFv
NPA4 binding with secreted scFv
NPA5 binding with secreted scFv
NPB1 binding with secreted scFv
O.D.450
3.5
3.0
2.5
2.0
1.5
1.0
0.5
0.0
0th
1st
2nd
3rd
& : The selected library panning phages used for 2nd antigen depletion panning

Supplementary Figure S3. The results of the antibody discovery procedure depicted in Figure 1B. (A) The output phage titer/input phage titer ratios (y-axis) for each of the phage display selection cycles (x-axis) against the 6 target NPs (as indicated in the panels) are plotted for each of the 16 phage-displayed synthetic antibody libraries (as indicated in the legends of the panels). The procedure of determining the phage titer is described in Methods. (B) The polyclonal scFv secretions in the culture media of the output phage-displayed libraries were measured with ELISA for the target NP (indicated in each panel) binding and the ELISA results are shown in the histogram for each of the 3 rounds of selection cycle (indicated by the color of the histogram in the legends of the panels). The histograms are plotted for each of the 16 phage-displayed synthetic antibody libraries (x-axis) in each of the panels in this figure. The synthetic antibody library design and construction have been described in Jian and Chen et al[1]. The arrows in red and blue indicate the output phage display libraries from second and third round phage display selection respectively, which were used as the input phage display libraries in the Step 2 of the procedure shown in Figure 1B. The experimental measurements for phage titer and polyclonal scFvs binding to the NPs with ELISA are described in Methods[1].

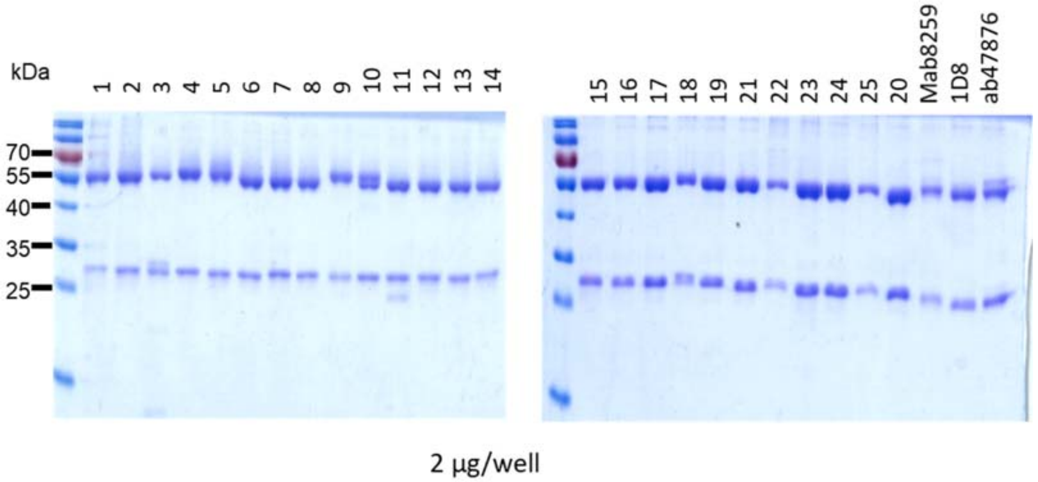

Supplementary Figure S4. SDS-PAGE analysis of the purified 25 anti-NP IgG1s. Three micrograms of each of the 25 purified anti-NP IgG1s along with the commercially available control positive antibodies were analyzed by the SDS-PAGE under reducing condition.

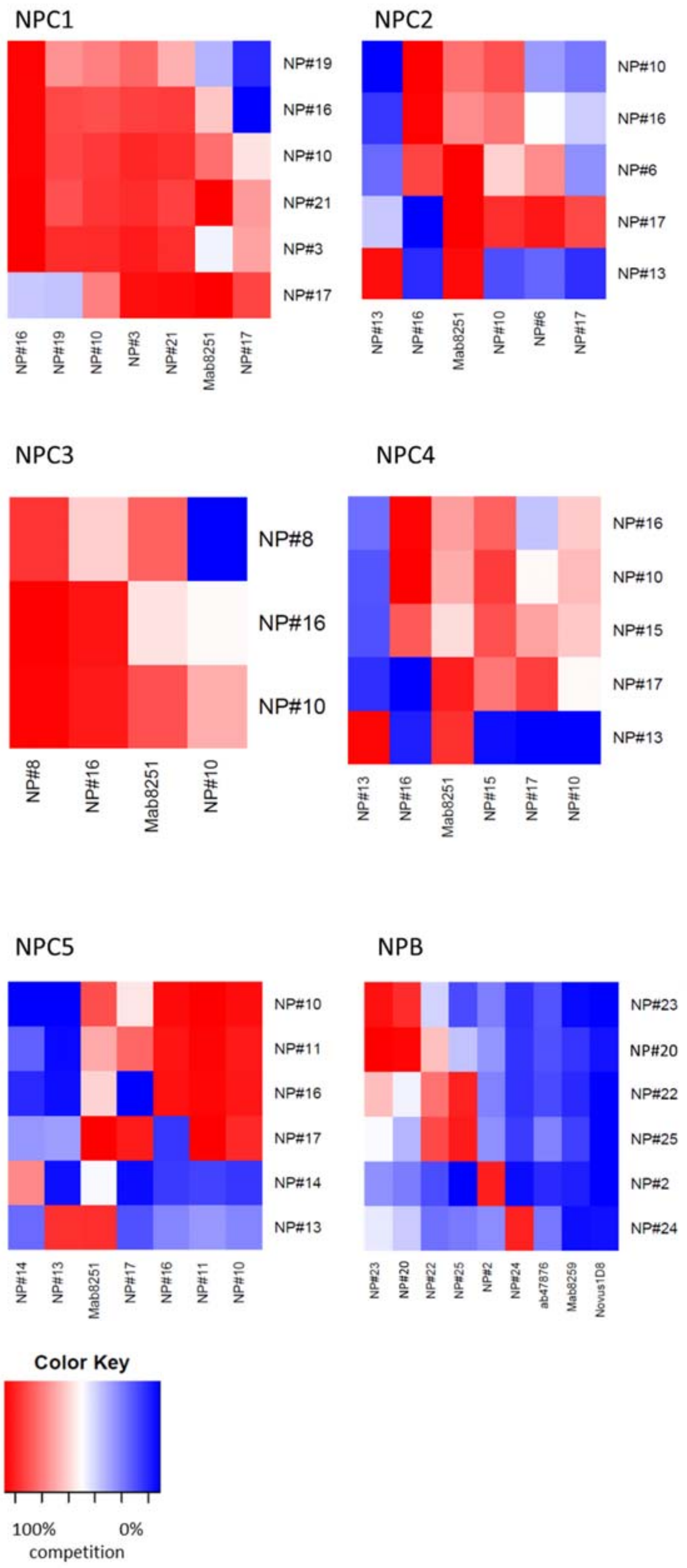
NPC1
NP#19
NP#16
NP#10
NP#21
NP#3
NP#17
NP#16 NP#19 NP#10 NP#3 NP#21 Mab8251 NP#17
NPC2
NP#10
NP#16
NP#6
NP#17
NP#13
NP#13 NP#16 Mab8251 NP#10 NP#6 NP#17
NPC3
NP#8
NP#16
NP#10
NP#8 NP#16 Mab8251 NP#10
NPC4
NP#16
NP#10
NP#15
NP#17
NP#13
NP#13 NP#16 Mab8251 NP#15 NP#17 NP#10
NPC5
NP#10
NP#11
NP#16
NP#17
NP#14
NP#13
NP#14 NP#13 Mab8251 NP#17 NP#16 NP#11 NP#10
NPB
NP#23
NP#20
NP#22
NP#25
NP#2
NP#24
NP#23 NP#20 NP#22 NP#25 NP#2 NP#24 ab47876 Mab8259 Novus1D8
Color Key
100%
0%
competition

Supplementary Figure S5. Competition of the anti-NP antibodies binding to the 6 representative NPs. The competition ELISA patterns are color-coded from blue to red with decreasing relative signal (the color scheme on the lower-left corner of the panel) resulting from the scFv (y-axis) binding to NP (as labelled on top of each panel) immobilized in the ELISA well in the presence of the competing IgG (x-axis). Experimental details are described in a previous publication [1].

	L1(L30~L32)	L2(L49~L53)	L3(L91~L96)	H1(K30~H33)	H2(H50~H58)	H3(H93~H102)	Len(H3)
NP1	DDD	SGATW	YFSWPI	NGGW	SIWPSGGSTY	ARWSSDY	7
NP2	YGY	YGAAG	YSNFPL	GGGG	GIWPYWGYTF	ARFWHGNLDVMDY	13
NP3	GWY	YSTTG	YYNYPI	SNYG	GIWPFGGFTS	ARFHWNDHGYMDY	13
NP4	GWG	SGPSS	YFNYPI	SNSS	GIGPSGGYTF	ARFHSHNSYYSYSHYGYMDY	20
NP5	GWS	YGSRW	GYNGPL	SNGS	GIWPSGGSTS	ARWNY	5
NP6	YFG	YGTSY	FSNWPI	NDGS	GIGPFGGFTY	ARSGYSGSFDY	11
NP7	FGW	SYSAS	YFNYPL	SNGG	GIGPYGGFTS	ARFYSNYYGYHGVMDY	16
NP8	DDS	YWTRY	GYNYPL	NSFG	GIGPFGGSTF	ARGFYWFDY	9
NP9	WFS	YGTTS	YFNYPL	NNGW	GIGPYWGFTS	ARFDSDHDHHYSYYYYYDNGYMDY	24
NP10	SWS	SGSTW	YSNSPI	DNGS	WIWPFGGSTS	ARSWSSGYDY	10
NP11	SSW	SWSGY	GFNYPL	DSYG	WIGPSGGSTF	ARGYFWFFDY	10
NP12	GGN	SSTAG	YYDFPL	DNGY	SIWPSWGSTY	ARGFYYYGDY	10
NP13	GYW	SSASG	GWDWPI	NNYG	SIGPYGGSTY	ARFGYFNWLDMDY	13
NP14	DND	YGPRG	YSSWPL	DNGG	SIWPSWGSTS	ARFNFGVFGVMDY	13
NP15	DNN	SYSTG	YWNYPI	SNWY	GIGPYGGSTY	ARFHWDHLHGMDY	13
NP16	GSY	SWTTG	YFSYPV	GNSG	WIGPYWGFTS	ARFGNHWHLFMDY	13
NP17	GYS	SSPGG	YYDYPV	SGGS	SIWPFGGSTF	ARFGHGHNVIMDY	13
NP18	GYY	YGPRF	YSNWPI	SGGG	SIWPSWGSTS	ARFHNGGFLVMDY	13
NP19	SGW	SWSTG	YFNFPV	SSWG	GIGPFWGYTS	ARFGVDNYGYMDY	13
NP20	GYG	SGASG	YSSFPL	NGYS	SIGPYGGFTY	AGFGFYY	7
NP21	YFY	YGSRW	YSNFPV	NGYG	GIWPYGGSTF	ARFYNNHGGIMDY	13
NP22	FSW	SSASS	SWDYPI	SDWS	GIWPYWGSTS	ARGFYSWFDY	10
NP23	DDG	SGARW	YYNFPL	SNYF	SIGPFGGFTS	AGYGFHY	7
NP24	GSS	FYTSS	GWDSPV	DDWY	GIGPYGGFTY	ARGYYGSFDY	10
NP25	SSG	SWTSF	YYDYPI	GGGF	SIWPYWGFTY	ARGSYGYSDY	10

Supplementary Table S1. The CDR sequences of the 25 representative scFvs indicated in Figure 2. The CDR residue positions in Kabat numbering are indicated in the top row. The antibody framework sequence has been described previously[1].

	NPA1	NPA2	NPA3	NPA4	NPA5	NPB1
NP1	NC					
NP2						1.37
NP3	0.09					
NP4	0.19		10.11		0.37	
NP5	1.69	0.13				
NP6						
NP7		0.79				
NP8		2.14	0.07			
NP9			4.38		1.12	
NP10	0.36		NC	NC	0.28	
NP11		0.27			0.06	
NP12					9.07	
NP13			9.36	0.07	0.09	
NP14	0.19	0.08	19.71		1.70	
NP15	NC	0.45		0.35		
NP16	0.09		0.96	0.18	0.09	
NP17	0.08	0.06		0.03	0.17	
NP18		0.20				
NP19	0.11	NC				
NP20						0.04
NP21	0.12	28.63				
NP22						0.27
NP23						0.06
NP24						0.05
NP25						0.10
MAB8251	0.12	0.13	0.13	3.68	0.16	NC
ab47876						0.13
NBP2-23514						0.11
MAB8259						0.27

Blank: No ELISA signals with 10 µg/ml IgG.

NC: curve fitting failed (Not converged, interrupted)

Supplementary Table S2. The EC_{50}'s (nM) derived from the sigmoidal binding curves of the 25 anti-NP IgG1s (first column from left) binding to the corresponding recombinant NP (first row from top) in Figure 3.

	A/California/07/2009 (H1N1/H1S)	A/Brisbane/59/2007 (H1N1/H1B)	A/Brisbane/10/2007 (H3N2/H3B)	A/Wisconsin/67/2005 (H3N2/H3W)	A/Vietnam/1194/2004 (H5N1/H5V)	B/Brisbane/60/2008 (fluB)
NP1	NC	NC	NC	NC	5.89	NC
NP2				NC	NC	4.89
NP3	0.55	0.521	0.301	0.25	0.393	NC
NP4	4.02	14.1	63.1	4.82	NC	NC
NP5	NC	NC	NC	NC	NC	NC
NP6	6.46	NC	NC	NC	NC	NC
NP7	NC	NC	NC		NC	NC
NP8			NC	NC	NC	
NP9	2.14	2.95	0.891	2.35	2.14	NC
NP10	NC	NC	NC		1	14.5
NP11	NC	NC			NC	
NP12	6.09	5.09	0.399	0.333	0.189	NC
NP13	1.677	NC	NC	5.633	7.17	NC
NP14	3.43	0.228	0.246	NC		NC
NP15	0.249	0.668	0.563	0.449	0.525	NC
NP16	0.239	0.21	0.21	0.248	0.501	NC
NP17	28.7	NC	NC		NC	NC
NP18	0.168	7	6.82	0.192	0.292	
NP19	0.304	0.295	0.39	0.215	0.386	NC
NP20	NC	NC	NC		NC	4.88
NP21	0.73	4.6	74.1	NC	13.7	NC
NP22		7.11	NC		NC	3.92
NP23	NC	NC	NC	NC	NC	
NP24				NC	NC	3.17
NP25	NC	4.83	NC	NC	NC	2.17
MAB8251	0.0813	0.08	0.131	0.085	0.316	NC
ab47876	NC		NC	NC	NC	2.06
NBP2-23514		NC	NC	NC		3.7
MAB8259	NC	NC			NC	9.09

Blank: No ELISA signals with 10 µg/ml IgG.

NC: curve fitting failed (Not converged, interrupted)

Supplementary Table S3. The EC_{50}'s (nM) derived from the sigmoidal binding curves of the 25 anti-NP IgG1s (first column from left) binding to the NPs in the influenza virus-infected MDCK cells (first row from top) in Figure 4.

(A)

	A/California/07/2009 (H1N1/H1S)	A/Brisbane/59/2007 (H1N1/H1B)	A/Brisbane/10/2007 (H3N2/H3B)	A/Wisconsin/67/2005 (H3N2/H3W)	A/Vietnam/1194/2004 (H5N1/H5V)	B/Brisbane/60/2008 (fluB)
NP1	NC	NC	NC	NC	NC	NC
NP2	NC	NC	NC	NC	NC	NC
NP3	1.955	2.36	3.433	1.812	2.015	NC
NP4	2.11	2.337	3.398	2.263	2.31	
NP5	3.321	4.02	5.496	3.128	3.498	NC
NP6	3.119	78.31	NC	2.696	3.037	
NP7	2.358	4.133	8.086	2.26	2.247	NC
NP8	2.328	NC	NC	2.602	2.636	
NP9	53.94	NC	NC	NC	NC	NC
NP10	3.297	3.967	4.592	3.202	3.376	
NP11	NC	NC	NC		NC	NC
NP12	NC	NC	NC	NC	NC	NC
NP13	168	135	59.29		54.1	
NP14	NC		NC	NC	NC	NC
NP15	2.789	4.608	6.221	2.525	2.815	
NP16	4.408	4.739	6.247	4.096	4.333	NC
NP17	3.277	4.083	4.406	3.087	3.251	
NP18	NC	NC	NC	NC	NC	NC
NP19	5.373	3.368	4.017	3.876	3.967	NC
NP20	NC	NC	NC	NC	NC	NC
NP21	2.671	2.71	3.535	2.882	2.791	
NP22	NC	59.44	NC	91.4	NC	NC
NP23		NC	NC	NC	NC	NC
NP24		NC	NC	NC	NC	
NP25	NC	NC	NC	NC	NC	

(B)

	A/California/07/2009 (H1N1/H1S)	A/Brisbane/59/2007 (H1N1/H1B)	A/Brisbane/10/2007 (H3N2/H3B)	A/Wisconsin/67/2005 (H3N2/H3W)	A/Vietnam/1194/2004 (H5N1/H5V)	B/Brisbane/60/2008 (fluB)
NP1	NC	NC	NC	NC	NC	NC
NP2	NC	NC	NC	NC	NC	NC
NP3	0.834	1.037	1.603	0.8276	0.8614	NC
NP4	0.8618	0.9107	1.288	0.867	0.8747	
NP5	0.9178	1.825	3.167	0.8789	0.909	
NP6	3.07	NC	NC	1.983	2.719	NC
NP7	0.8998	4.152	5.861	0.8206	0.8792	
NP8	0.7521	NC	NC	0.8545	0.9226	NC
NP9	NC	NC	NC	106.3	NC	NC
NP10	0.7976	0.8732	1.058	0.8422	0.8602	NC
NP11	NC	NC	NC	NC	NC	NC
NP12	NC	NC	NC	NC	NC	NC
NP13	47.88	27.87	11.03	35.78	28.95	NC
NP14	NC	NC	NC	NC	NC	
NP15	0.8056	0.926	1.133	0.7792	0.7976	NC
NP16	0.9193	0.89	0.9594	0.891	0.8361	NC
NP17	1.024	2.218	3.333	1.072	1.195	
NP18	NC	66.44	1000	1000	1000	NC
NP19	0.8634	0.8117	0.9426	0.7655	0.8141	NC
NP20	NC	NC	NC	NC	NC	NC
NP21	0.815	0.81	1.062	0.8475	0.8791	NC
NP22	NC	102.4	98.22	82.84	NC	NC
NP23	NC	NC	NC	NC	NC	NC
NP24	NC	NC	NC	NC	NC	NC
NP25	NC	NC	NC	NC	NC	NC

Blank: No ELISA signals with 10 µg/ml NP.

NC: curve fitting failed (Not converged, interrupted)

Supplementary Table S4. The EC_{50}'s (nM) of the virus NPs (first row from top) derived from the sigmoidal binding curves in Figure 5 of the sandwich ELISAs with HRP-conjugated NP16 (Table S4A) and NP17 (Table S4B) as detection antibody and the 25 anti-NP IgG1s (first column from left) as capture antibodies.

References

1. Jian JW, Chen HS, Chiu YK, Peng HP, Tung CP, Chen IC, et al. Effective binding to protein antigens by antibodies from antibody libraries designed with enhanced protein recognition propensities. MAbs 2019; 11:373-87.
